# Ultrasonic control of neurite outgrowth direction

**DOI:** 10.1038/s41598-021-99711-0

**Published:** 2021-10-11

**Authors:** Haruki Maruyama, Koji Fujiwara, Masahiro Kumeta, Daisuke Koyama

**Affiliations:** 1 Faculty of Science and Engineering, Doshisha University, 1-3 TataraMiyakodani, Kyotanabe, Kyoto, 610-0321 Japan; 2 Graduate School of Biostudies, Kyoto University, Yoshida Konoe-cho, Sakyo-ku, Kyoto, 606-8501 Japan

**Keywords:** Biomedical engineering, Cell growth, Applied physics

## Abstract

This study investigated a method to control neurite outgrowth direction using ultrasound vibration. An ultrasound cell culture dish comprising a glass-bottom culture surface and a glass disc with an ultrasound transducer was fabricated, and undifferentiated neuron-like PC12 cells were grown on the dish as an adherent culture. The 78 kHz resonant concentric flexural vibration mode of the dish was used to quantitatively evaluate the neurite outgrowth direction and length. Time-lapse imaging of cells was performed for 72 h under ultrasound excitation. Unsonicated neurites grew in random directions, whereas neurite outgrowth was circumferentially oriented during ultrasonication in a power-dependent manner. The neurite orientation correlated with the spatial gradient of the ultrasound vibration, implying that neurites tend to grow in directions along which the vibrational amplitude does not change. Ultrasonication with 30 V_pp_ for 72 h increased the neurite length by 99.7% compared with that observed in unsonicated cells.

## Introduction

In vitro experiments using cultured cells can evaluate their response to various factors, such as chemicals, stress, and temperature. In vivo environments are important because they focus not only on a single cell but also communication among neighboring cells. Cells within living tissues such as muscles and blood vessels have suitable periodic structures and orientations (for example, vascular endothelial cells are aligned in the direction of blood flow). Therefore, the positional and directional relationships among cells is important and should be controlled precisely in vitro to quantitatively evaluate cell communication. Numerous techniques for cell manipulation have been reported. In particular, the “optical tweezer” method is a powerful tool for noncontact manipulation of cells^1,2^. Although this optical method enables high-precision positioning of a single cell, a high-intensity optical beam is required, and it is difficult to control a cluster of cells. Instead, cell manipulations using microchannel^3–5^ and chemical^6^ techniques may be used.

Cells are randomly oriented in a cell culture environment in the absence of stimuli. To simulate in vivo conditions, the position and orientation of cells can be controlled using electric fields^7,8^, microfabrication techniques^9–12^, and optical methods^1,13^. The electric field method exploits the polarity of cells to simultaneously manipulate a large number of them over a long distance in the electric field; however, the experimental setup is bulky and its application is limited to specific types of cells, such as nerve cells. Microfabrication techniques form biomimetic polymer scaffolds on the bottom of culture dishes to direct cellular adhesion. Although this technique can be applied to various types of adhesive cells, the fabrication process is costly considering the disposability of culture dishes. In addition, direct fabrication on the surface of culture dishes poses a contamination risk. Using optical methods, the direction of cells can be precisely controlled by the light distribution, although it is difficult to downsize the experimental setup.

Ultrasound is a promising tool for cell manipulation. In an ultrasonic standing wave, objects much smaller than the vibrational wavelength can be trapped at nodal or anti-nodal positions^14–17^. By controlling the acoustic standing-wave temporally and spatially, small objects can be transported without physical contact. The size of objects that can be manipulated by ultrasound depends on the wavelength, with smaller objects being trapped using higher frequencies. Cells with a size of tens of micrometers can be trapped and manipulated using ultrasound in the MHz to tens of MHz range (wavelength of tens to hundreds of micrometers in water). Interdigital transducers on piezoelectric substrates can be used for cell manipulation because they easily radiate high intensity and frequency surface acoustic waves^18–22^. Although this technique is suitable for single-cell manipulation and precise positioning can be achieved, it is difficult to align the orientation of cells in one direction across a wide surface area. Kurashina et al. have reported a technique for cultivating adhesive cells using ultrasound vibration^23^ and showed that the resonant flexural vibration of the cell culture surface combined with chemical treatment improved the cultivation efficiency of the cells. Cheng et al. reported the patterning of neural cells in hydrogel scaffolds using a three-dimensional ultrasonic standing wave^24^, showing the direction of neurites can be aligned along the nodal plane.

Our previous work^25^ reported an adhesive cell patterning technique using ultrasound vibrations. The positions where HeLa cells could adhere depended on the vibrational distribution across the culture dish, and large ultrasound vibration inhibited the cell growth although the viability of the cells was not affected. The position where HeLa cells grow could therefore be controlled by the flexural vibration of the dish, although the direction of cellular outgrowth was not addressed. Our research group has developed a method to control the molecular orientation of liquid crystals on glass substrates using ultrasound vibration^26,27^. Although the sizes of biological cells are 3–4 orders of magnitude larger than liquid crystal molecules, these results suggest this method could be used to control cell orientation. In this study, a method to control the orientation of adhesive cells using ultrasound was investigated. Low-frequency (tens of kHz) ultrasound with a wavelength exceeding that of the surface acoustic wave was used to control the cells across a wide surface area of the culture dish.

## Materials and methods

**Figure 1:**
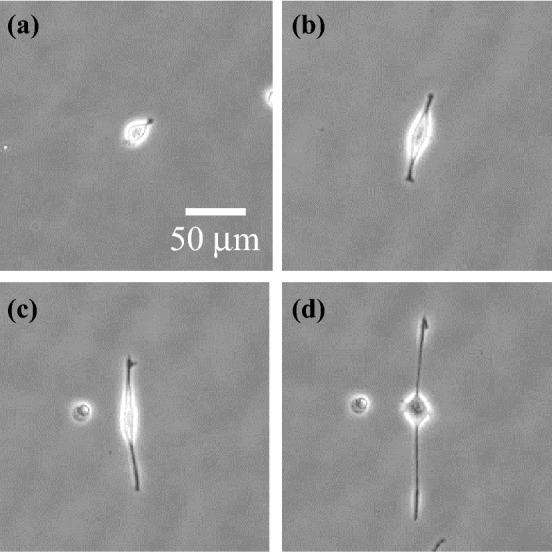
Phase-contrast images of the neurite growth from a PC12 cell after adding nerve growth factor (NGF). Images were acquired at (**a**) day 1, (**b**) day 2, (**c**) day 3, and (**d**) day 4.

In this study, the effects of ultrasound vibration on the morphology and orientation of adhesive cells were investigated. Adhesive cells grow by attaching to a culture dish, and their growth gradually declines as they approach confluence. Proliferating cells were plated on new culture dishes and maintained using standard subculture techniques. Undifferentiated neuron-like PC12 cells were purchased from the Institute of Physical and Chemical Research, Japan, and grown as an adherent culture in Dulbecco’s modified eagle medium (DMEM, Wako Pure Chemical Industries, Osaka, Japan) supplemented with 10% v/v horse serum (Lot No. 2147675, Thermo Fisher Scientific, MA), 10% v/v fetal bovine serum (Lot No. 015BS493, Wako Pure Chemical Industries, Osaka, Japan), and 1% v/v penicillin/streptomycin (Wako Pure Chemical Industries, Osaka, Japan). The cells were incubated at 37 °C in a 5% CO_2_ atmosphere and serially subcultured once per week. The culture medium was removed from the culture dish and the cells were washed with Dulbecco’s phosphate-buffered saline (Nacalai Tesque, Kyoto, Japan). One milliliter of trypsin solution (Nacalai Tesque, Kyoto, Japan) was added to release the adhered cells, then fresh culture medium was added to buffer the cytotoxicity of trypsin. Ten milliliters of the resulting cell suspension was centrifuged, and the cell pellet was resuspended in fresh culture medium and transferred to new culture dishes. To evaluate of the effects of ultrasound vibration on neurite outgrowth, nerve growth factor (NGF) was added after the subculture to differentiate the PC12 cells into neuron-like cells and induce neurite outgrowth. The subcultured PC12 cells were immersed in DMEM supplemented with 50 ng/mL NGF, 10% v/v horse serum, 10% v/v fetal bovine serum, and 1% v/v penicillin/streptomycin. Figure 1 shows images of the neurite outgrowth from a PC12 cell 1–4 days after adding NGF.

**Figure 2:**
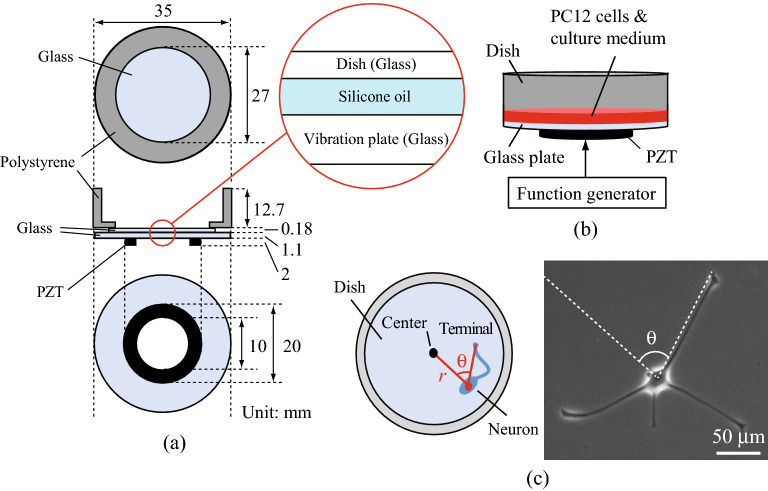
(**a**) Configuration of the ultrasound cell culture dish^25^. (**b**) Experimental setup. *PZT* piezoelectric lead zirconate titanate. (**c**) Definitions of radial position (*r*) and growth direction (*θ*) of neurites on the dish. The growth direction (*θ*) was defined by the angle between the center of the dish, the center of the soma, and the terminus of the longest neurite.

An ultrasound cell culture dish developed in our previous work^25^ was used (Fig. 2). An annular piezoelectric lead zirconate titanate transducer (inner diameter: 10 mm; outer diameter: 20 mm; thickness: 1 mm; C-213, Fuji Ceramics, Fujinomiya, Japan) polarized in the thickness direction was glued to a circular glass plate (diameter: 35 mm; thickness: 1.1 mm). This vibrating glass disc was in contact with the bottom of a polystyrene cell culture dish (outer diameter: 35 mm; inner diameter: 27 mm; AGC Techno Glass, Shizuoka, Japan) having a glass bottom (diameter: 30 mm; thickness: 0.18 mm) to excite the ultrasound vibrations on the bottom of the dish. Considering the disposability of the culture dish and the acoustic impedance matching between the glass dish and the vibrating glass disc (acoustic impedance: ~ 1.3 × 10^7^ Pa·s/m), silicone oil (~ 9.5 × 10^5^ Pa·s/m) was introduced as a coupling agent between the dish and the vibration plate so that the ultrasound vibrations could be transferred efficiently to the bottom of the dish. This configuration helps to prevent contamination because the ultrasound transducer is not in contact with the culture medium and cells. The sizes of the piezoelectric ring and the glass plate required to efficiently generate tens-of-kHz resonant flexural vibration modes on the bottom of the dish were numerically determined using the finite element method.

The subcultured PC12 cells were grown with the initial cell density of 3 × 10^5^ cells/mL in the ultrasound cell culture dish with 2 mL of culture medium, resulting in a liquid depth of approximately 2 mm. The viability assay using propidium iodide (PI) solution (Cosmo Bio, Tokyo, Japan) was conducted, and the ratio of dead cell before ultrasonication was approximately 6%. The dish was mounted in a small chamber in which the temperature, humidity, and CO_2_ gas density were controlled. A continuous sinusoidal electric signal from a function generator was input to the piezoelectric ring to generate ultrasound flexural vibrations on the bottom of the dish 20 h after seeding (*t* = 0). The experimental setup is comparatively inexpensive since the ultrasound dish has a simple structure and costly equipment and micromachining processes are not required. Time-lapse images of the plated cells within a 10 × 10 mm^2^ area were obtained using an inverted fluorescence microscope (IX83, Olympus, Tokyo, Japan) for 72 h under ultrasound excitation. The images were taken at *t* = 0, 1, 4, 24, 28, 48, 52, and 72 h. To evaluate the neurite outgrowth direction, the distance, *r*, of the cell from the center of the dish was measured, and the growth direction of the neurite was defined by the angle, *θ*, between the center of the dish, the center of the soma, and the terminus of the longest neurite, as shown in Fig. 2c. The center of the soma was determined from the brightness distribution of the microscopic image; the darkest point corresponds to the center of the soma. The neurite length was also measured to investigate the effects of ultrasound on neuronal differentiation. Five hundred cells were measured using commercial image analysis software (Cell Sens, Olympus, Tokyo, Japan) under the condition that both the center of the soma and the neurite terminus were not overlapped with other cells. The vibrational distribution across a central 27 × 27 mm^2^ area of the dish surface was measured using a laser Doppler vibrometer (LDV, NLV-2500, PI Polytec, Waldbronn, Germany), and the vibrational displacement amplitude on the dish can be measured from the vibration velocity.

Mechanical stress caused by vibrational impact affects gene expression of cells^28–30^. The effect of ultrasound vibration on the gene expression profile of PC12 cells was investigated using real-time quantitative PCR analysis. Total RNA from PC12 cells was extracted using an RNeasy kit (74104, Qiagen, Hilden, Germany) before and after 72 h of ultrasonication. Reverse transcription-coupled real-time quantitative PCR was performed using a One-step SYBR PrimeScript Plus RT-PCR kit (RR096B, Takara Bio, Shiga, Japan) and a LightCycler 480 RT-qPCR system (Roche Diagnostics, Basel, Switzerland). The expression level of synapsin-I (*Synl*) and adenosine A2a receptor (Adora2a) genes that are respectively activated and inactivated during neuronal differentiation^31,32^ were measured under each driving condition. The relative mRNA levels of these genes were calculated relative to the expression level of the ribosomal protein L29 (*RPL29*) gene that is stable during the neuronal differentiation^33^. The expression level of ribosomal protein L19 (*RPL19*) gene was also analyzed as a second reference gene. Analyses were performed three times with triplicated measurements. Statistical significance among samples was evaluated by one-way ANOVA followed by Tukey’s honestly significant difference (HSD) test and indicated in each graph. The following probe sets were used to detect each target gene: *SynI* (forward) 5′-ggacggaagggatcacatta-3′, (reverse) 5′-tggtgatccccaatgagtg-3′/*Adora2a* (forward) ccgaattccactccggta, (reverse) gttcccgtctttctgactgc/*RPL19* (forward) tgccggaagaacaccttg, (reverse) gcaggatcctcatccttcg/*RPL29* (forward) ttgccaagaagcacaacaag, (reverse) ggcatcttgggcttgaca.

## Results and discussion

**Figure 3:**
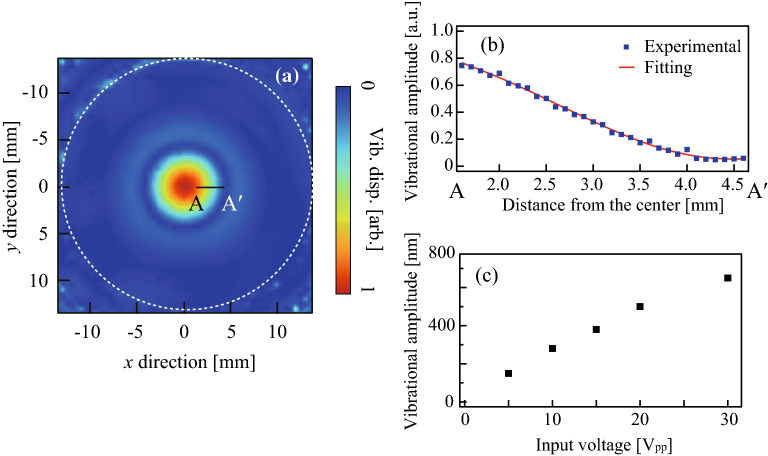
Vibrational characteristics of the ultrasound cell culture dish at 78 kHz measured by the LDV. (**a**) Distribution of the vibrational displacement amplitude across the bottom of the dish. A dotted circle indicates the border of the glass bottom of the cell culture dish. (**b**) Radial dependence of the vibrational amplitude along the line from A to A′ shown in (**a**). (**c**) Relationship between the input voltage to the ultrasound transducer and the vibrational displacement amplitude. The vibrational displacement amplitude in panels (**a,b**) was normalized relative to its maximum value.

To investigate the relationship between the vibrational distribution on the ultrasound culture dish and the growth direction of neurites, we analyzed one of the axisymmetric resonance vibration modes of the dish between 20 and 100 kHz. Figure 3a shows the out-of-plane vibrational displacement amplitude distribution across the bottom of the dish at a resonance frequency of 78 kHz. The vibrational distribution across the dish was measured in the absence of culture medium since the reflected light from the medium surface interfered with the observed signal. The vibrational displacement amplitude was largest at the center of the dish, and an axisymmetric resonant flexural vibration mode with concentric circular nodes and no nodal line was generated. The radius of the smallest node is approximately 4.5 mm, which is suitable for the microscopic observation (Fig. 3b). The solid red line indicates the fitting curve using the theory for the flexural vibration of a disc^34^. The vibrational amplitude was proportional to the input voltage to the ultrasound transducer, and the maximum out-of-plane vibrational amplitudes at the center of the plate and the ultrasound transducer were 0.65 μm and 0.09 μm, respectively, when the input voltage and electric power to the transducer were 30 V_pp_ and 0.34 W, respectively (Fig. 3c).

**Figure 4:**
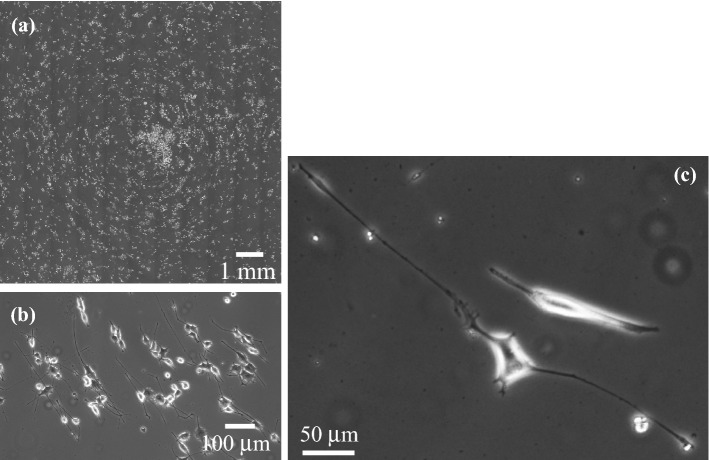
Phase-contrast images of PC12 cells at *t* = 72 h (92 h after adding the NGF) under 78 kHz ultrasonication with the transducer input voltage of 30 V_pp_.

**Figure 5:**
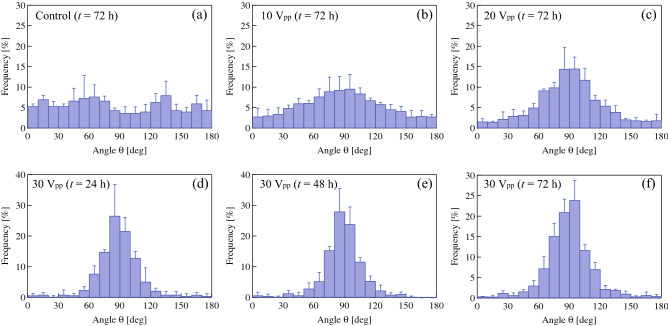
Frequency distributions of the growth directions of neurites excited with 78 kHz ultrasound using transducer input voltages of (**a**) 0 (control), (**b**) 10, (**c**) 20, (**f**) 30 V_pp_ at *t* = 72 h (n = 500), and 30 V_pp_ at (**d**) *t* = 24 (n = 86) and (**e**) 48 h (n = 172). The angles of 0° (or 180°) and 90° correspond to the radial and tangential directions on the culture dish, respectively. Data represent the mean and standard deviation for three measurements. The growth directions were measured using microscopy under the condition that both the center of the soma and the neurite terminus were not overlapped with other cells.

Figure 4 shows the phase-contrast image of PC12 cells after 72 h of ultrasound excitation. A uniform distribution of cells was observed because they had adhered to the bottom of the dish prior to beginning the ultrasound excitation (ultrasonication prior to cell adhesion causes aggregation in response to the acoustic radiation force, leading to cell patterning^25^). The image shows that most neurites grew parallel to the circumference of the dish. The effect of the vibrational amplitude on the growth direction of neurites was also investigated. Figure 5 shows the frequency distributions of the growth direction with different input voltages to the ultrasound transducer. The growth directions were distributed randomly in the absence of ultrasonication (Fig. 5a), whereas tangential neurite growth (*θ* = 90°) was evident in the presence of ultrasound excitation (Fig. 5b–f). Tangential growth was enhanced by increasing the input voltage (Fig. 5b,c,f) and changed little with time (Fig. 5d–f), indicating that the growth direction of the neurites is precisely controlled by ultrasound vibration. In the cases without (control) and with ultrasonication (30 V_pp_), the cell densities increased by 130% and 122%, respectively, for 72 h, implying that the ultrasound vibration under the experimental conditions did not significantly affect the cell density and the cells were not detached by ultrasound vibration on the culture dish.

**Figure 6:**
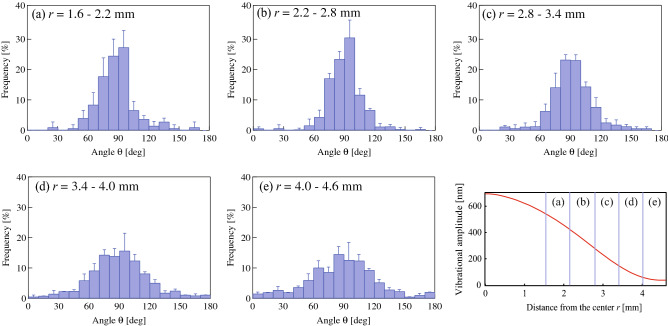
Frequency distributions of neurite growth directions at *t* = 72 h in annular regions (**a**) 1.6–2.2 mm, (**b**) 2.2–2.8 mm, (**c**) 2.8–3.4 mm, (**d**) 3.4–4.0 mm, and (**e**) 4.0–4.6 mm from the center of the dish excited with the input voltage of 30 V_pp_ at 78 kHz. 100 cells were measured in each region using microscopy under the condition that both the center of the soma and the neurite terminus were not overlapped with other cells. Data represent the mean and standard deviation for three measurements. Points at *r* = 0 mm and *r* = 4.5 mm correspond to the vibrational loop and nodal positions, respectively (bottom right).

**Figure 7:**
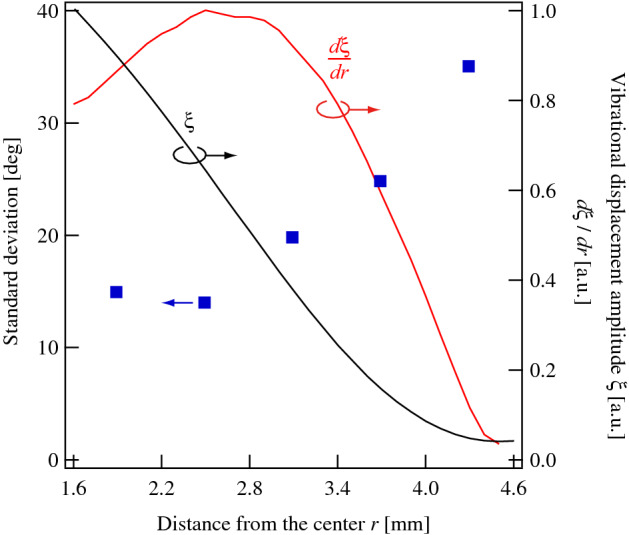
Radial dependence of the standard deviation of the neurite outgrowth orientation (blue), vibrational displacement amplitude *ξ* (black), and spatial gradient of the vibrational amplitude *dξ*/*dr* (red) on the culture dish excited with 30 V_pp_ at 78 kHz at *t* = 72 h. The standard deviations were calculated from the results in Fig. 6.

To investigate the radial dependence of the results and evaluate the growth direction quantitatively, the observational area on the dish was divided into five annular regions between *r* = 1.6 and *r* = 4.6 mm (Fig. 6). The center of the dish (*r* < 1.6 mm) was excluded from the observational area because the radius of curvature was small, making the definition of growth direction difficult to measure in circular coordinates. Figure 6 shows the radial dependence of the growth direction of neurites at a transducer voltage of 30 V_pp_. The vibrational distribution across the dish and in the different annular regions was also investigated. The growth direction of neurites in each region was close to *θ* = 90°, although the orientational dispersion varied between them. The orientational dispersion of neurite outgrowth was evaluated assuming that the growth directions in each region follow a normal distribution. Figure 7 compares the radial dependence of the standard deviation of the growth direction, the vibrational displacement amplitude, *ξ*, and the spatial gradient of the displacement amplitude, *dξ*/*dr*. Similar to the results in Fig. 5, a larger vibrational amplitude resulted in a smaller standard deviation. The smallest standard deviation was obtained in the annular region between *r* = 2.2 and *r* = 2.8 mm where the mean and standard deviation of the growth direction were 92.5° and 13.9°, respectively. This result indicates that the growth direction depends not only on the vibrational amplitude at the position of the cell body but also on the surrounding area. Accordingly, the growth direction correlates more strongly with *dξ*/*dr* than with *ξ*. Similar behavior was observed for the molecular orientation of liquid crystals by acoustic radiation force^35^, although the sizes of the objects were different. Using microfabrication techniques, the neurite outgrowth directions can be aligned along micropatterns, such as those formed by microchannels^36^, implying neurite terminals respond to topographic changes in the surrounding area and change their growth direction accordingly. These findings suggest that neurite outgrowth occurs along a direction where the vibrational amplitude does not change and that this effect is enhanced by using larger spatial gradients of the vibrational displacement amplitude.

**Figure 8:**
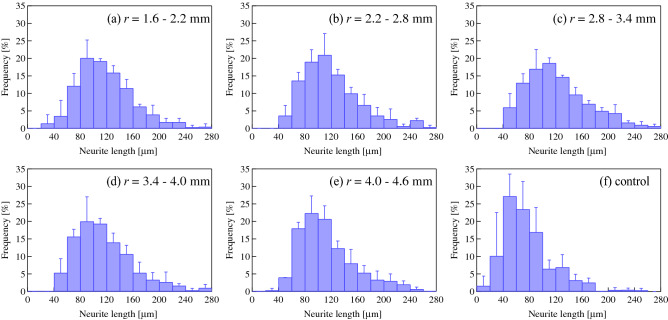
Frequency distributions of the neurite lengths at *t* = 72 h in annular regions (**a**) 1.6–2.2 mm, (**b**) 2.2–2.8 mm, (**c**) 2.8–3.4 mm, (**d**) 3.4–4.0 mm, and (**e**) 4.0–4.6 mm from the center of the dish excited with 30 V_pp_ at 78 kHz. (**a**–**e**) 100 and (**f**) 500 cells were measured using microscopy under the condition that both the center of the soma and the neurite terminus were not overlapped with other cells. Data represent the mean and standard deviation for three measurements.

**Figure 9:**
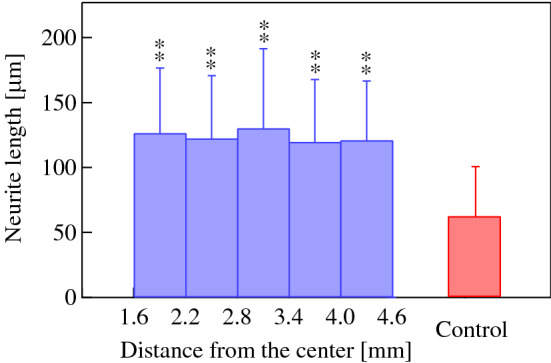
Average neurite lengths at *t* = 72 h in annular regions between 1.6–2.2, 2.2–2.8, 2.8–3.4, 3.4–4.0, and 4.0–4.6 from the center of the dish excited with 30 V_pp_ at 78 kHz. Control: unsonicated PC12 cells. 500 sonicated (blue) and unsonicated (red) cells were measured using microscopy under the condition that both the center of the soma and the neurite terminus were not overlapped with other cells. Data represent the mean and standard deviation calculated from the results in Fig. 8. Statistical significance among samples was evaluated by one-way ANOVA followed by Tukey’s HSD test and indicated in each graph. **Indicates *p* < 0.01 to the control (*F* = 58.3).

**Figure 10:**
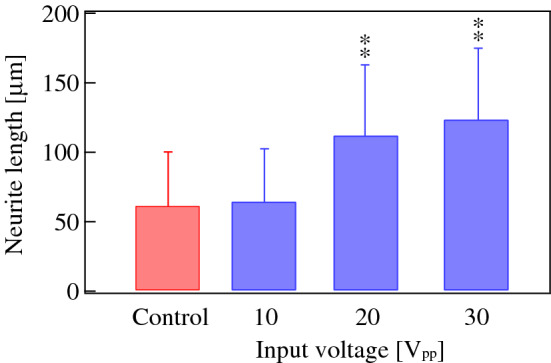
Relationship between the transducer input voltage and neurite length 72 h after ultrasonication at 78 kHz. 500 cells in an annular region at *r* = 1.6–4.6 mm from the center of the dish were measured under each condition under the condition that both the center of the soma and the neurite terminus were not overlapped with other cells. Data represent the mean and standard deviation. Statistical significance among samples was evaluated by one-way ANOVA followed by Tukey’s HSD test and indicated in each graph. **Indicates *p* < 0.01 to the control (*F* = 255).

The effect of ultrasonication on neurite length was also investigated. Neurite lengths in different annular regions were measured after 72 h of 30 V_pp_ ultrasound excitation. Figure 8 shows the distributions of the neurite lengths in each region. The mean and standard deviation of the neurite lengths in each region were calculated assuming they follow a normal distribution (Fig. 9). Although the neurite length did not depend on the radial position of cells between *r* = 1.6 and *r* = 4.6 mm, cells that were ultrasonicated grew neurites that were twice as long as those that were unsonicated (control) (*p* < 0.01 (*F* = 58.3) Tukey’s test). The average neurite length in the annular region from *r* = 4.0 to *r* = 4.6 mm was significantly larger than that of the control, although the vibrational amplitude was almost zero in both cases, which implies that the ultrasound vibration in the area surrounding the cells affects the neurite outgrowth. Figure 10 shows that the average neurite length increased as a function of the ultrasound transducer input voltage, indicating that a larger vibrational displacement amplitude of the glass substrate excited with the input voltage over 20 V_pp_ (the maximum displacement amplitude of 0.43 μm) promotes neuronal differentiation.

**Figure 11:**
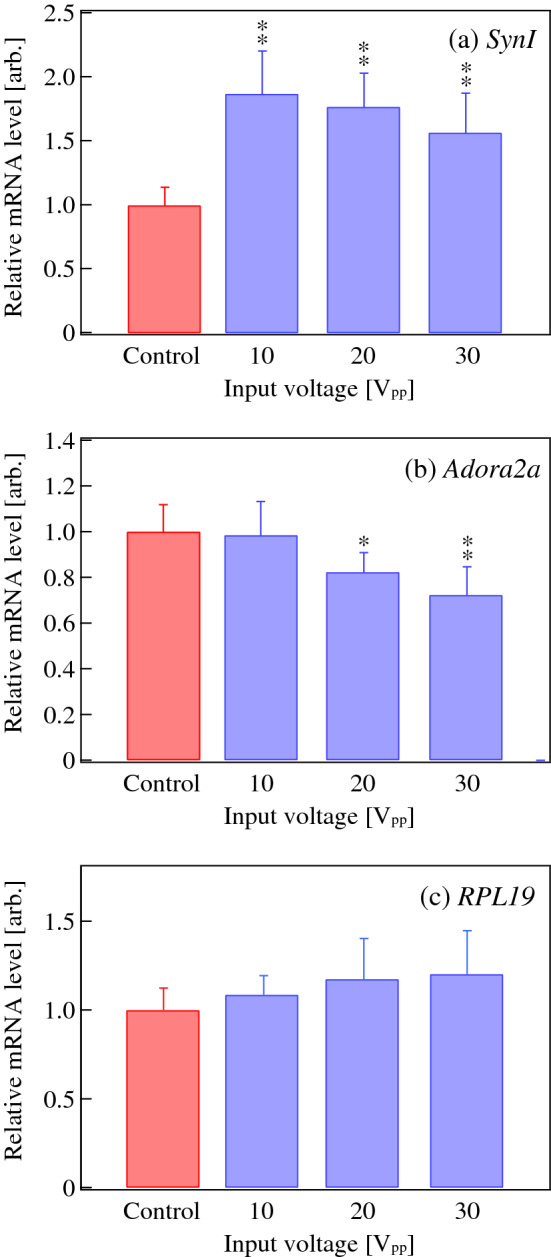
The relative mRNA levels of (**a**) synapsin-I (*SynI*), (**b**) adenosine A2a receptor (*Adrora2a*), and (**c**) a reference gene ribosomal protein L19 (*RPL19*) on the ultrasound transducer input voltage. The analyses were performed three times with triplicated measurements. Control: unsonicated PC12 cells. Statistical significance among samples was evaluated by one-way ANOVA followed by Tukey’s HSD test and indicated in each graph. *Indicates *p* < 0.05 and **indicates *p* < 0.01 to the control (*F* = (**a**) 16.37, (**b**) 9.60, and (**c**) 1.93).

To assess the effect of ultrasound vibration on the neuronal differentiation of PC12 cells, changes in gene expression profiles were investigated. Neurite outgrowth triggered by NGF is known to accompany alterations in a set of differentiation-related genes in PC12 cells, including activation of the synaptogenesis regulator synapsin-I (*SynI*) and suppression of the adenosine A2a receptor gene (*Adora2a*). Figure 11a,b show the mRNA levels of *Synl* and *Adora2a* relative to a reference gene ribosomal protein L29 (*RPL29*) in unsonicated cells (control) and in cells sonicated using input voltages from 10 to 30 V_pp_ for 72 h. The result of another reference gene ribosomal protein L19 (*RPL19*) is also shown in Fig. 11c, which remained constant. Compared with the control condition, ultrasound excitation induced a statistically significant activation of *SynI* and suppression of *Adora2a*, indicating that neuronal differentiation is promoted by ultrasound stimulation. A larger suppression of *Adora2a* was observed in response to larger input voltages, which correlates with the larger neurite outgrowth observed using microscopy (Fig. 10).

Ultrasonic modulation of neuronal activities has been reported using hippocampal slice culture and ex vivo mouse brain. Transmission of low-frequency (0.44–0.67 MHz) and low-intensity ultrasound activated voltage-gated sodium and calcium channels and triggered synaptic transmission by activating SNARE-mediated exocytosis of synaptic vesicles^37^. Cell-level impact of ultrasound stimulation on neuritogenesis has also been revealed by using rat dorsal root ganglion in monoculture or co-cultured with Schwann cells^38,39^. Application of 0.5 MHz pulsed ultrasound on these cultured cells significantly induced outgrowth, length, and branching of the neurites. Interestingly, secreted factors from Schwann cells subjected to ultrasound stimulation seemed to be a major contributor for neuronal modulation in this case, rather than the calcium signaling^39^. Therefore, there may be multiple mechanisms in ultrasound-sensitive neuronal modulation, including both calcium-sensitive and insensitive ones. Neuritogenesis of PC12 cells has been known as highly sensitive to calcium signaling^40,41^. Considering the above-mentioned effect of ultrasound on sodium and calcium channels, ultrasonic stimulation applied in this study might trigger calcium signaling that led to the activated neuritogenesis. Further studies focusing on the pathways controlling neurite formation will uncover the mechanism underlying ultrasonic control of neurite in PC12 cells.

In this paper, the neurite length and orientation could be controlled by ultrasound vibrations under particular conditions using the cell line. Although ultrasound in hundreds kHz to MHz range is often used for cell stimulation^37,42^, the experiments in this paper were performed using low-frequency (78 kHz) continuous ultrasound because the wavelength of the ultrasound vibration is suitable for the microscopic observation to find the relationship between the vibrational distribution of the culture dish and the neurite outgrowth direction. In addition, the ultrasound vibration with the displacement amplitude under 0.65 μm that did not damage and scrub the cells on the culture dish was used. The technique to control the orientation of living tissue would be a powerful tool in regenerative medicine. For instance, the orientation relationship between the coating of implants and bone tissue largely affects the healing period and the bone strength^43^.

## Conclusions

A method to control the neurite outgrowth direction using ultrasound vibration was evaluated. The direction and length of neurite outgrowths from cells grown on the surface of an ultrasound cell culture dish were quantitatively evaluated. The outgrowths were oriented circumferentially, along which directions the ultrasound vibrational amplitude was constant. This effect was enhanced by using larger input voltages to the ultrasound transducer and the orientational dispersion was concomitantly decreased. Ultrasonication increased the neurite length by 99.7% compared with that observed in unsonicated cells, indicating that the neurite length and orientation can be controlled by ultrasound vibrations on a cell culture dish. In addition, ultrasonication induced a statistically significant activation of *SynI* and suppression of *Adora2a*, indicating that neuronal differentiation is promoted by ultrasound stimulation.
